# Supplementary material for: Pregestational neurological disorders among women of childbearing age—Nationwide data from a 13-year period in Hungary
Source: PLoS One. 2022 Sep 21;17(9):e0274873. doi: 10.1371/journal.pone.0274873 (PMC9491540; doi:10.1371/journal.pone.0274873)
Supplement: Attachment — Submitted filename: Response to Reviewers.docx [file pone.0274873.s003.docx]

**János Szentágothai Doctoral School of Neurosciences**

Semmelweis University

Chairman: Prof. Dániel Bereczki MD. PhD. DHAS

H-1085 Budapest, Üllői út 26.

Tel.: +36-1-459-1500

Tel.: +36-1-459-1500

Budapest, June 9, 2021

**Dr. D. M. Basavarajaiah**

**Academic Editor**

**Public Library of Science One**

Dear Editor,

thank you for the valuable feedback provided on our manuscript Bereczki D Jr. et al: **Pregestational neurological disorders among women of childbearing age – nationwide data from a 13-year period in Hungary** submitted with the intention to publish it in the journal **PLOS ONE**.

Please, find our responses to the reviewers’ comments:

***“(i)The author unable to describe the research gap and rationality is not up to the mark, the practical utility of the research is well planned and derived properly. In Methodological section, I have not seen , what are the tests used for assessing the patients and selection of concomitant variables.”***

The relevant part of the manuscript in the “Introduction” part on describing the research gap and rationality has been updated as follows:

*“Comprehensive statistics from large population on neurological disorders affecting future pregnancies are scarce, thus besides concentrating on specific diseases among women in general or focusing on conditions developing during gestation itself, it seems rational to retrospectively study those women of childbearing age who have certainly become pregnant and had a delivery and thereby evaluate the magnitude of pregnancies complicated already at the time of conception by various neurological diseases. Nationwide data on such selected population would be desirable to further optimize prenatal care from the very beginning of gestation and thereby improve maternal and fetal outcomes.”*

The “Materials and methods” section of the manuscript has been divided into two main parts: “Database design” and “Data evaluation”. In the latter part, it is now emphasized that we have used descriptive statistics by inserting the following sentence to the very beginning of the section:

*“During data analysis, we used descriptive statistics.”*

Also, for the sake of clarity, we updated the wording in two sentences:

1. Instead of *“For the identification and classification of neurological and obstetrical conditions, three-digit codes from the 10^th^ International Classification of Diseases (ICD-10) were used.”,* the updating wording is as follows: *“For the identification of labours and for the classification of neurological disorders, three-digit codes from the 10^th^ International Classification of Diseases (ICD-10) were applied.”*

2. Instead of *“Temporal distribution of obstetrical and neurological diagnoses enabled the identification (…)”*, the updated wording is as follows: *“Temporal distribution of the receipt of labor-related and neurological diagnoses given by clinical specialty areas enabled the identification (…)”*.

Also, in the “Materials and methods” section, the ethics statement has been updated emphasizing that this was a retrospective study of medical records and also approval number provided by the Ethics Committee of Semmelweis University, Budapest, Hungary was added. The updated wording is as follows: *“During the retrospective study of medical records, data anonymization was provided by the use of encrypted codes derived from original patient identifiers, thereby also making record linkage possible. Study approval was provided by the Ethics Committee of Semmelweis University, Budapest, Hungary (Approval No.: SE TUKEB 88-1/2015) and data management was in line with personal data protection rules.”*

***“(ii) In result part, the research hypothesis is not tested by relevant statistical methods .The flow of resulted part is not fulfilled our journal criteria. Plz repharse the sentence and describe the result part by using accurate estimation of variables to be tested by statistical methods.”***

As pointed out above and also emphasized in the revised manuscript, we used descriptive (and not comparative) statistics during our work. As per SAMPL guidelines, we updated the “Results” part by providing numerators and denominators to all percentages.

***“(iii) Discussion and conclusion part is not fulfilled our journal criteria”***

The “Discussion” part has been split into three subunits with following titles: “Pregestational neurological disorders in general”; “Specific neurological disorders before conception”; “Strengths and limitations”. The subunit “Specific neurological disorders before conception” has been further divided into subsections.

The “Conclusion” part has been reworded putting more emphasis on the research gap. The updated wording is as follows: *“As the scientific literature is scarce on data from large populations concerning the wide range of neurological disorders, nationwide statistics enabling the optimization of prenatal care are desirable.”*

As per request, funding statement has been amended and included in the updated cover letter as follows: “The work was supported by the National Brain Research Program (2017-2-1-NKP-2017-00002). There was no additional external funding received for this study. The funders had no role in study design, data collection and analysis, decision to publish, or preparation of the manuscript.”

We hope that after considering our revisions and comments, you will find our manuscript worth to consider it for publication.

Sincerely yours,

Dániel Bereczki Jr., MD

Corresponding author

E-mail: bereczki.daniel@hotmail.com
