# Supplementary material for: Pregestational neurological disorders among women of childbearing age—Nationwide data from a 13-year period in Hungary
Source: PLoS One. 2022 Sep 21;17(9):e0274873. doi: 10.1371/journal.pone.0274873 (PMC9491540; doi:10.1371/journal.pone.0274873)
Supplement: Attachment — Submitted filename: Revised Manuscript with Track Changes (Autosaved).docx [file pone.0274873.s004.docx]

**Pregestationalneurological Disorders among Women of Child bearing age – Nationwide data from a 13-year period in Hungary**

DánielBereczki Jr.^1^*, Mónika Bálint^2^, András Ajtay^3,4^, Ferenc Oberfrank^5^, Ildikó Vastagh^3,4,6^

^1^János Szentágothai Doctoral School of Neurosciences, Semmelweis University, Budapest, Hungary

^2^Centre for Economic and Regional Studies, Budapest, Hungary

^3^Department of Neurology, Semmelweis University, Budapest, Hungary

^4^MTA-SE Neuroepidemiological Research Group, ELKH, Budapest, Hungary

^5^Institute of Experimental Medicine, Budapest, Hungary

^6^Department of Neurology, Bajcsy-Zsilinszky Hospital and Clinics, Budapest, Hungary

*Corresponding author

Email: bereczki.daniel@hotmail.com (DB)

**Abstract**

Objectives

Comprehensive statistics evaluating pregnancies complicated by various medical conditions are desirable for the optimization of prenatal care and for improving maternal and fetal outcomes.Our main objective of study was to assess pregnancies during a 13-year study period with accompanying pregestational neurological disorders in medical history on a nationwide level.

Methods

In the framework of the NEUROHUN 2004-2017 project utilizing medical reports submitted for reimbursement purposes to the National Health Insurance Fund, we included women with at least one laborduring (2004-2016) who had at least one pregestationaldiagnosis of a neurological disorder received within this time frame prior to their first pregnancy during the studied period. Three-digit codes from the 10^th^ International Classification of Diseases (ICD) were used for the identification and classification of neurological and obstetrical conditions.The data sets were collected in occrdance with standard operating protocol of the ICD .

Results

A specific inclusion and exclusion criteria was employed during the study process . A total 744 226 women have been identified with at least one delivery during the study period with 98792 of them (13.3%) having at least one neurological diagnosis received during 2004-2016 before their first gestation in the time frame of the study . The vast majority of diagnosis codes were related to different types of headaches affecting 69149 (9.29%) individuals. The most prevalent diagnoses following headaches were dizziness and giddiness (15 589 patients (2.09%); nerve, nerve root and plexus disorders (10 375 patients-1.39%); epileptic disorders (7028 patients 0.94%); neurological diseases of vascular origin (6091 patients 0.81%); other disorders of the nervous system (5358 patients 0.71%); and demyelinating diseases of the central nervous system (2129 patients 0.28%). The present findings of our study shows high prevalence of pregestational neurological disorders, the massive dominance of headaches followed by the rather nonspecific diagnosis of dizziness and giddiness, the relevance of nerve, nerve root and plexus disorders and epilepsy, and the importance of cerebrovascular disorders amongwomen of childbearing age.

Conclusion

Present research findings will help the neurologists , researchers and policy makers for implmenetation of helath policy and escalates to develop new algorithms of various neurological manifestations at larger extent.

Key words : ???

**Introduction**

Management of pregnancies accompanied by neurological disorders can be a complex medical challenge necessitating tight follow-up and multidisciplinary approach[affix suitable reference]. The relationship between gravidity and neurological conditions is bilateral [affix suitable reference]. On the one hand, physiological changes during gestation can modify the course of certain diseases [affix suitable reference]. For example, in pregnancy, migraine without aura can improve or recedein over (70%) of the patients **[1]**. Gravidity has a disease modifying effect on multiple sclerosis (MS) with a relative reduction of relapse rates in the third semester followed by a rebound in the postpartum period **[2]**. In a study assessing the course of the disease in pregnancy, (50%) of pregnant women with myasthenia gravis (MG) showed a deterioration (mainly during the second trimester), while (30%) reported an improvement of the symptoms **[3]**. While the majority of epileptic women have a seizure controlsimilar to that of the pregestational baseline, (17.3%) experience an increase, while (15.9%) encounter a decrease in seizure frequency **[4]**. Furthermore, pregnancy and puerperium present three fold higher incidencerates for ischemic and hemorrhagic strokes as compared with nonpregnant women **[5]**. On the other hand, neurological diseases can also have an impact on pregnancy outcomes either *per se* or indirectly, *i.e.* bytheir medication or via relevant diagnostic procedures affecting fetal development [affix suitable reference]. For example, patients with myasthenia gravis, epilepsy, obstructive sleep apnea or acute migraine are at increased risk of complications during gestation and/or delivery **[6-9]**. Compared with the general population, womensuffering astroke or TIA (TIA full form) at fertile age show higher rates of miscarriages orfetal death throughout their lives **[10]**. Potential detrimental consequencesof fetal exposure to certain anti-epileptic, anti-MS(MS full form) and anti-MG (MG full form) drugs have been documented **[6,11-13]**. Comprehensive statistics of large scale population on neurological disorders affecting future pregnancies are scarce, thus besides concentrating on specific diseases among women in general or focusing on conditions developing during gestation itself, it seems rational to retrospectively study those women of childbearing age who have certainly become pregnant and had a delivery and thereby evaluate the magnitude of pregnancies complicated already at the time of conception by various neurological diseases.Due to paucity of literature we have to assess the pregnancies during a 13-year study period with accompanying pregestational neurological disorders in medical history by utilizing nation wide data on selected attributes of neurological traits .In this context of the research gap , we optimize the prenatal care from the varying gestation period to explore the fudicial gap of the research intervention by using massive data sets [13]. In the era of big data, digital medical records can underlie such databases, particularly in countries with a single-payer state health insurance system covering the whole population **[14]**. In this nationwide epidemiological study, our aim was to explore and assess the first pregnancies in a 13-year period with different neurological diagnoses.

**Materials and methods**

**Database design and source data for evaluation**

the neurohun 2004-2017 database **[14]** was created within the scope of the hungarian national brain research program (NBRP) from medical reports submitted for reimbursement purposes to the national health insurance fund (NHIF) from all hospitals and specialist outpatient services throughout the country. furthermore, records regarding demographic and socioeconomic factors were obtained from the hungarian central statistical office (HCSo) . the full massive databank covered a 14-year period between (2004 - 2017). in the present analysis, we included women with at least one laborduring (2004-2016) who had at least one pregestationaldiagnosis of a neurological disorder receivedwithin this time intervalprior to their first pregnancy during the study period. to exclude non-clinical specialty areas (e.g. laboratory diagnostics, diagnostic imaging, physiotherapy, psychology, *etc*.), only diagnoses were confirmed by clinical specialties , have been involved in the study by the use of specific clinical specialty codesapplied in hungary. it is to be noted, that reports submitted by general practitioners werenot included in the database. During data analysis, we used descriptive statistics. For the identification of labors and for the classification of neurological disorders, three-digit codes from the 10^th^ International Classification of Diseases (ICD_10_) **[15]**wereapplied. Conditions deemed as“neurological”were determined by the study team and comprised the following diagnostic groups(with corresponding ICD~~10~~ codes):

# Malignant neoplasms of eye, brain and other parts of central nervous system (C_69_–C_72_)

# [Benign neoplasm](https://en.wikipedia.org/wiki/Benign_neoplasm) of [meninges](https://en.wikipedia.org/wiki/Meninges) ([D](http://apps.who.int/classifications/icd10/browse/2016/en#/D32)_[32](http://apps.who.int/classifications/icd10/browse/2016/en#/D32)_)

- [Benign neoplasm](https://en.wikipedia.org/wiki/Benign_neoplasm) of [brain](https://en.wikipedia.org/wiki/Human_brain) and other parts of [central nervous system](https://en.wikipedia.org/wiki/Central_nervous_system) ([D](http://apps.who.int/classifications/icd10/browse/2016/en#/D33)_[33](http://apps.who.int/classifications/icd10/browse/2016/en#/D33)_)
- [Neoplasm](https://en.wikipedia.org/wiki/Neoplasm) of uncertain or unknown behavior of [meninges](https://en.wikipedia.org/wiki/Meninges) ([D](http://apps.who.int/classifications/icd10/browse/2016/en#/D42)_[42](http://apps.who.int/classifications/icd10/browse/2016/en#/D42)_)
- [Neoplasm](https://en.wikipedia.org/wiki/Neoplasm) of uncertain or unknown behavior of [brain](https://en.wikipedia.org/wiki/Human_brain) and [central nervous system](https://en.wikipedia.org/wiki/Central_nervous_system) ([D](http://apps.who.int/classifications/icd10/browse/2016/en#/D43)_[43](http://apps.who.int/classifications/icd10/browse/2016/en#/D43)_)
- Diseases of the nervous system ([G](http://apps.who.int/classifications/icd10/browse/2016/en#/D43)_[00](http://apps.who.int/classifications/icd10/browse/2016/en#/D43)_[-G](http://apps.who.int/classifications/icd10/browse/2016/en#/D43)_[99](http://apps.who.int/classifications/icd10/browse/2016/en#/D43)_)
- Cerebrovascular diseases ([I](https://www.icd10data.com/ICD10CM/Codes/I00-I99/I60-I69)_[60](https://www.icd10data.com/ICD10CM/Codes/I00-I99/I60-I69)_[-I](https://www.icd10data.com/ICD10CM/Codes/I00-I99/I60-I69)_[69](https://www.icd10data.com/ICD10CM/Codes/I00-I99/I60-I69)_)
- Dizziness and giddiness (R_42_)
- Headache (R_51_)

The number of deliveriesduring (2004-2016) were assessed by the application ofthe labor-related codes O_60_ (“Preterm labor and delivery”) and O_80_-O_84_ (“Delivery”) from ICD_10_given by any clinical specialties during inpatient service. The “Delivery” groupcomprised the codes “Single spontaneous delivery” (O_80_), “Single delivery by forceps and vacuum extractor” (O_81_), “Single delivery by caesarean section” (O_82_), “Other assisted single delivery” (O_83_), and “Multiple delivery” (O_84_).Temporal distribution of the receipt of labor-related and neurological diagnoses given by clinical specialty areasenabled the identification of those patients, who were diagnosed with a neurological condition during the studied years prior to their first pregnancyinthe study period as in these cases, the date of the neurological diagnosis preceded the labor-related ICD-_10_ code by more than nine months. Hence, those women with a sole delivery between (2004-2016) occurring in the first 9 months of the 13-year study periodwere excluded from the present analysis.During the retrospective study of medical records, data anonymization was provided by the use of encrypted codes derived from original patient identifiers, thereby also making record linkage possible. Study approval was provided by the Ethics Committee of Semmelweis University, Budapest, Hungary (Approval No.: SE TUKEB 88-1/2015) and data management was in line with personal data protection rules. Primary data acquisition was performed by a research assistant with IT specialization, with profound experience in studying medical records of patients. For the final analysis, results of individual searches in the database were exported to excel files used for further evaluation during the final analysis.

**Results**

**The general prevalence of pregestational neurological disorders**

By the use of the abovementioned inclusion and exclusion criteria, 744 226 women have been identified with at least one delivery during the study period.Out of them, thosehaving at least one neurological diagnosis received during the 13-year time framebefore their first pregnancy in the studied interval resulted in 98 792 cases .Thus, (13.3%) ([98 792/744 226] x 100)of abovementioned 744 226 women had already received at least one neurological diagnosis during 2004-2016prior to their first gestationin the study period and became pregnant with that in mind. (Table 1) shows the number of patients classified by neurological diagnoses received during the 13-year time frame before their first pregnancy within the study interval. As the majority of these ICD-10 codes represent diagnostic groups, patients receiving more than one ICD _10_ code within one diagnostic group were counted only once (the number of all diagnoses without filtering repetitions are presented in parentheses). Nevertheless, individual women could appear in multiple differentdiagnostic categories.(Table 2) shows detailed data on patient numbers for “Episodic and paroxysmal disorders” (G_40_-G_47_).

**Table 1. Classification of patients by neurological diagnoses received in (2004-2016) prior to first pregnancy during the study period**

| **ICD_10_ numerical code** | **Diagnostic Classification** | **Number of patients (Number of diagnoses, ifapplicable)** |
| --- | --- | --- |
| C_69_-C_72_ | Malignantneoplasms of eye, brain and otherparts of centralnervoussystem | 256 [263 –(%)] |
| D_32_-D_33_ | Benignneoplasm of meninges (D_32_) Benignneoplasm of brain and otherparts of centralnervoussystem (D_33_) | 721 [ (756-(%)] |
| D_42_-D_43_ | Neoplasm of uncertainorunknownbehaviour of meninges (D_42_) Neoplasm of uncertainorunknownbehaviour of brain and centralnervoussystem (D_43_) | 257 [ (259-(%)] |
| G_00_-G_09_ | Inflammatorydiseases of thecentralnervoussystem | 423 [ (481-(%)] |
| G_10_-G_14_ | Systemicatrophiesprimarilyaffectingthecentralnervoussystem | 66 [ (67-(%)] |
| G_20_-G_26_ | Extrapyramidal and movementdisorders | 1551 [ (1606-(%))] |
| G_30_-G_32_ | Otherdegenerativediseases of thenervoussystem | 180 [ (180-(%)] |
| G_35_-G_37_ | Demyelinatingdiseases of thecentralnervoussystem | 2129 [ (2381-(%)] |
| G_40_-G_47_ | Episodic and paroxysmaldisorders | 40814 [ (46712-(%)] |
| G_50_-G_59_ | Nerve, nerveroot and plexusdisorders | 10375 [ (10877-(%)] |
| G_60_-G_64_ | Polyneuropathies and otherdisorders of theperipheralnervoussystem | 1107 [ (1194-(%)] |
| G_70_-G_73_ | Diseases of myoneuraljunction and muscle | 655 [ (722-(%)] |
| G_80_-G_83_ | Cerebralpalsy and otherparalyticsyndromes | 738 [ (872-(%)] |
| G_90_-G_99_ | Otherdisorders of thenervoussystem | 5358 [ 5598-(%] |
| I_60_-I_69_ | Cerebrovasculardiseases | 3082 [ 3669-(%)] |
| R_42_ | Dizziness and giddiness | 15589 -(%) |
| R_51_ | Headache | 51096-(%) |

**Table 2. Number of patients with G_40_-G_4_7 diagnoses received in (2004-2016) prior to first pregnancy during the study period**

| **ICD _10 numerical_ code** | **Diagnostic cassification** | **Number of patients (Number of diagnoses, ifapplicable)** |
| --- | --- | --- |
| G_40_-G_41_ | Epilepsy (G_40_) Status epilepticus (G_41_) | 7028 [7089-%] |
| G_43_ | Migraine | 12909[%] |
| G_44_ | Otherheadachesyndromes | 21086[%] |
| G_45_ | Transientcerebralischaemicattacks and relatedsyndromes | 3508[%] |
| G_46_ | Vascularsyndromes of brainincerebrovasculardiseases | 63[%] |
| G_47_ | Sleepdisorders | 2057[%] |

**The prevalence of specific pregestational neurological disorders**

(i)**Headaches (G_43_-G_44_; R_51_)**

Analysis of the data demonstrated a massive dominance of diagnoses referring to different types of headaches. This group of ICD-_10_ codes containing “Migraine” (G_43_; 12 909 (% ) cases ), “Other headache syndromes” (G_4_4; 21 086 (% ) cases ), and “Headache” (R_51_; 51 096 cases ) added up a total of 85 091 diagnoses. Nevertheless, as individual women could appear in more types of headache categories, such overlaps were excluded resulting in 69149 patients. Notably, the abovementioned individuals displayed (70%) ([69 149/98 792] x 100) of women with at least one neurological diagnosis received between 2004 and 2016 prior to their first pregnancy during the studied years, and (9.3%) ([69149/744 226] x 100) of all women with at least one labor in the study period.

(ii) **Dizziness and giddiness (R_42_)**

In terms of prevalence, different types of headaches were followed by the diagnosis of “Dizziness and giddiness” (R_42_) affecting 15 589 cases , thereby making this category, the second most common pregestational neurological diagnosis with (15.8% ) ([15 589/98 792] x 100) and comprised of (2.1%) ([15 589/744 226] x 100) of all individuals with at least one delivery during the study period.

(iii) **Nerve, nerve root and plexus disorders (G_50_-G_59_)**

Total (10.5% ) affecting ([10 375/98 792] x 100) of women with at least one neurological diagnosis received during (2004-2016) before their first pregnancy in the study period, “Nerve, nerve root and plexus disorders” (G_50_-G_59_; 10 375 cases ) ranked third among the top ICD _10_ categories. This group involved (1.4% )([10 375/744 226] x 100) of all women with at least one labor during the studied years. Within this diagnostic interval,“**Mononeuropathies of upper limb” (G_56_) were the most represented disorders with (28.5%) (**[2953/10 375] x 100) **affecting 2953 cases.Other relevant subgroups were “Disorders of trigeminal nerve” (G_50_) with (19.4%) (**[2013/10 375] x 100) **involving 2013 patients, “Nerve root and plexus disorders” (G_54_) with (19.3%) (**[2006/10 375] x 100) **comprising 2006 patients, “Facial nerve disorders” (G_51_) with (17.5%) (**[1818/10 375] x 100) **concerning 1818 cases and “Other mononeuropathies” (G_58_) with (11.2%) (**[1161/10 375] x 100) **affecting total of 1161 cases cases in the data sets**

# (iv)Epilepsy and status epilepticus (G_40_-G_41_)

# The G_40_-G_41_ diagnostic groupincluding “Epilepsy” (G_40_) and “Status epilepticus” (G_41_) involved 7028 women, comprising (7.1%) ([7028/98 792] x 100) of those with a pregestational neurological diagnosisprior to their first gestation during the study interval. Ofall women with at least one labor during (2004-2016) , (0.9%) ([7028/744 226] x 100) cases were received the diagnosis of epilepsy and/or status epilepticus during this time period prior to their first pregnancywithin the studied years.

**v) Cerebrovascular disorders (G_45_-G_46_; I_60_-I_69_)**

Although the ICD _10_ system classifies the category “Transient ischemic attacks and related syndromes” (G_45_) and “Vascular syndromes of brain in cerebrovascular diseases”(G_46_) separately, it seems reasonableto handle them along with “Cerebrovascular diseases” (I_60_-I69) when assessing neurological diseases of vascular origin. These 6091 patients displayed (6.2%)([6091/98 792] x 100)of women with at least one labor and a pregestationalneurological diagnosisprior to their first pregnancy between 2004 and 2016 and represented (0.8% )([6091/744 226] x 100) of all individuals with at least one deliveryduring the studied time frame, making cerebrovascular disorders almost as prevalent as epilepsy among these women of childbearing age. Within the category of cerebrovascular disorders, “Transient ischemic attacks and related syndromes” were the most represented (3508 cases), followed by diseases of the I_60_-I_69_ group displayed in (Fig 1) . The main representatives of this latter ICD-_10_ group were “Other cerebrovascular diseases” (I_67_; 1172 cases ),“Cerebral infarction” (I_63_; 803 cases ) and “Occlusion and stenosis of precerebral arteries, not resulting in cerebral infarction” (I_65_; 740 patients).

**Fig 1. Diagnoses of “Cerebrovascular diseases” (I-numerical codes) received during (2004-2016) prior to first pregnancy within the study period**

(i) **Other disorders of the nervous system (G_90_-G_99_)**

This ICD-_10_ category containing mainly unspecific neurological disordersranked among the most prevalent categories (5358 patients; 5598 diagnoses) owing to its subgroups “**Disorders of autonomic nervous system” (G_90_) with (51.5%)** ([2762/5358] x 100)**affecting 2762 cases patients and “Other disorders of brain” (G_93_) with (37.9%)** ([2031/5358] x 100) **including2031cases .**

(ii) **Demyelinating disorders of the central nervous system (G_35_-G_37_)**

This diagnostic interval of 2129 cases (2381diagnoses) was mostly represented by “Multiple sclerosis” (G_35_) with 82.2% ([1751/2129] x 100) affecting1751 patientsand displaying a prevalence of (0.2%) ([1751/744 226] x 100) among all women with at least one deliveryduringthe study time frame.

(iii) **Other neurological disorders**

In terms of prevalence, the beforementioned ICD-_10_ categories were followed by “Sleep disorders” (G_47_; 2057 cases ); “Extrapyramidal and movement disorders” (G_20_-G_26_; 1551cases ) and“Polyneuropathies and other disorders of the peripheral nervous system” (G_60_-G_64_; 1107 cases ). Further diagnostic groups comprising less women are presented in Table 1.

**Discussion**

In the present study, our objective was to assess the magnitude of first pregnancies of a 13-year study period with previous neurological diagnoses received during the studied years on a nationwide level, by deriving data from the NEUROHUN 2004-2017 project. The NEUROHUN database utilizing medical reports submitted for reimbursement purposes from a single-payer health insurance system covering the whole population has proven valuable in characterizing epidemiological features of various neurological conditions, such as ischemic stroke, headache, Parkinson’s disease or multiple sclerosis **[16-20]**.

(**i)Pregestational neurological disorders in general**

First, the totalnumber of women with at least one labor between 2004 and 2016 has been determined by the use of labor specific ICD-_10_ codes (O_60_; O_80_-O_84_) and by applying certain exclusion criteria. Out of these women, those with at least one neurological diagnosis received throughout this time period prior to their first pregnancy during the studied years were selected for further analysis. Based on the results, it has been revealed that (13.3%) of the 744 226 studied women had their first pregnancy within thegiven time interval already possessing at least one pregestationalneurological diagnosis received duringthe studyperiod. This can be considered a surprisingly high prevalence especially for sucha narrowly-defined population.

**(ii)Specific neurological disorders before conception**

a) **Headaches**

The vast majority of “neurological” ICD_10_ codes were related to different types of headaches with 85 091 diagnoses affecting 69 149individuals resulting in a prevalence of (70%) among the studied women having at least one neurological diagnosis received prior to their first pregnancy during 2004-2016and affecting 9.3% of those with at least one delivery in the study period. The high volume of these diagnoses was in correspondence with the results of the Global Burden of Disease Study 2016 with tension-type headache and migraine ranking among the top ten causes with the greatest prevalence worldwide**[21]**. Notably, headaches aremost burdensome in women of childbearing age (between ages 15 and 49 years) making 11.2% of all years of life lived with disability (YLD) in this age group and sex **[22]**.It should be taken into account, especially when comparing the abovementioned numbers to previous data showing a global prevalence of current headache being 47% **[23]**,that prevalence values of this study are based on medical reports representing only individuals seeking specialist medical care in response to their symptoms with records submitted by general practitioners being not included.It is also to be noted that among the 69 149 headache, (73.9%) ([51 096/69 149] x 100) received at least the nonspecific R_51_code“Headache”. This could partially be attributed to the fact, that as per study design, neurological diagnoses could have been given by any kind of clinical specialties with the possibility that non-neurologists tend to make no slight distinctions between different headache types during documentation.

(iii)**Dizziness and giddiness**

In terms of prevalence, although far behind headaches, the rather nonspecific diagnosis of “Dizziness and giddiness” (R_42_) ranked second among the most common neurological diagnoses in medical history with 15 589 patients. According to internal medicine outpatient service data, among leading complaints, dizziness is the third most common general symptom **[24]**.There are estimations regarding the lifetime prevalence of dizziness being 15-35% in the general population **[25]**.It is to be noted, that the etiological spectrum of dizziness is considerably wide, including the following top diagnostic groups: otologic/vestibular, cardiovascular, respiratory, neurologic (including cerebrovascular), metabolic, injury/poisoning, psychiatric, digestive, genitourinary, and infectious **[26]**.Furthermore, assessments regarding prevalence need to be evaluated cautiously as several different types of complaints can be described as “dizziness” by the patient (e.g. vertigo, disequilibrium, faintness, visual and gait disturbances, anxiety).When interpreting the relatively low prevalence in the current study with (2.1%) of women with at least one delivery during the study period being affected, it has to be taken into account that more specific diagnoses underlying this general complaint (e.g. benign paroxysmal vertigo, syncope and collapse, abnormalities of gait and mobility, diplopia, phobic anxiety disorders) were not included in this category.

(iv) **Nerve, nerve root and plexus disorders**

Within the third most common pregestational neurological diagnostic category of“Nerve, nerve root and plexus disorders” (G_50_-G59) affecting 10375 cases , “Mononeuropathies of the upper limb” (G_56_) were the most represented **involving lesions of the median, ulnar and radial nerves**. With an estimated lifetime risk of 10%, affecting women more likely than men and having an increasing incidence with age, carpal tunnel syndrome is the most common focal, compressive neuropathy of the upper extremity, followed by ulnar neuropathy due to entrapment in the elbow region **[27-30]**. Radial neuropathies generally can be caused either by external nerve compression (Saturday night palsy) or by trauma usually associated with fracture of the humerus**[27,29]**.Mononeuropathies of the upper limb were followed by “Disorders of trigeminal nerve” (G_50_) representing (19.4%) of nerve, nerve root and plexus disorders. It is to be noted that oro-facial pain has a high prevalence among women (30%) with the age group 18-25 years being the most affected **[31]**.The ICD-_10_ subgroup G_50_ involvesatypical facial pain, an underdiagnosed, debilitating condition with poor prognosis affecting most likely women in their forties. **[32]**.Being in this subgroup, trigeminalneuralgia also has the highest prevalence among women older than 40 years **[33]**, however, it may also need to be taken into account that multiple sclerosis being the most prevalent in the sex and age group of our study population **[34]**is associated with a 20-fold higher prevalence of trigeminal neuralgia **[35]**.Closely following disorders of the trigeminal nerve, “Ne**rve root and plexus disorders” (G54) displayed19.3% of diagnoseswithin this diagnostic interval (G50-G59). This could be explained by the fact, that these conditions can be etiologically related to neck pain and low back pain, the two main causes of disability of musculoskeletal origin [21].**These complaints ranked globally as the fourth leading cause of disability-adjusted life years (_DALY_) following ischemic heart disease, cerebrovascular disease, and lower respiratory infection **[36]**.**According to the Global Burden of DiseaseStudy 2016, low back pain was the leading cause of age-standardized YLD rates for women in most countries [21].**“Facial nerve disorders” (G_51_) ranked fourthamong nerve, nerve root and plexus disorders with (17.5%) of the diagnoses. Most well-known condition of this category is Bell’s palsy displaying the highest incidence between the ages of 15 and 45 years and having a complete recovery in 80% of non-pregnant women in this age group **[37]**.Notably, peripheral facial nerve palsy occurring during gestation has a much worse prognosis with only 61% of patients recovering fully**[37]**.Affecting 11.2% of patients within abovementioned diagnostic interval (G_50_-G_59_), “Other mononeuropathies” (G_58_) including e.g. intercostal neuropathy and mononeuritis multiplex, was the fifth most prevalent diagnosis among nerve, nerve root and plexus disorders.

(**v)Epileptic disorders**

Ranking after headaches, dizziness and giddiness, and nerve, nerve root and plexus disorders, epileptic disorders involving “Epilepsy” (G_40_) and “Status epilepticus” (G_41_) involved 7 028 patients of the study population. Although data are partly conflicting in this regard, it seems that in comparison to women without epilepsy, women with epilepsy over 25 years of age have significantly lower birth rates **[38]**. This fact may have several causes, partially attributed to the potential side effects of the antiepileptic treatment (influence on hormone levels and thereby sexual function; fear of fetal malformations) but psychosocial factors, psychiatric comorbidities or the fear of seizures during pregnancy may also take part in the above observation **[38]**. Nevertheless, according to recent data, women with epilepsy seeking pregnancy have a similar likelihood of getting pregnant and similar live birthrates when compared to women without epilepsy **[39]**. The prevalence of the diagnosis of epileptic disorders being 0.9% among women with at least one delivery in the given time period corresponds to the data on epilepsy affecting almost 1% of the population **[40]**.

(**vi)Cerebrovascular disorders**

According to literature data, 16 to 59 per 100 000 women of childbearing age are affected by stroke per year**[10,41]**.Surprisingly, neurological diseases of vascular origin affecting 6091 women in our study population ranked among the most prevalent neurological disorders. The prevalence of 0.8% among all women having at least onelaborduringthe study period points out the relevance of cerebrovascular disorders among women of childbearing age. This high prevalence can be attributed to the followings: (1) Age is a well-known non-modifiable cardiovascular risk factor **[42]**. During the last decades, especially in developed countries, there has been a dramatic increase in the number of women giving birth at an advanced age. In the United States, between 2007 and 2016, births rates have risen 11% for women in their late thirties and (19%) for women in their early forties**[43]**. Also, (20%) of babies born in England and Wales in 2013 had mothers aged 35 and over at the time of birth **[44]**.With advancing age, the increasing prevalence of different chronic conditions should also need to be taken into account. (2) The diagnoses of cerebrovascular disorders were given by any clinical specialists, including non-neurologists, with the possibility of assessing many – especially transient – symptoms as being of vascular origin. The latter hypothesis is supported by the relatively high prevalence of “TIA and related disorders”(G_45_) and by the rather aspecific group of “Other cerebrovascular diseases”(I_67_) being the most prevalent category within the I60-I69 interval. Nevertheless, it should be noted that the latter category includesdiagnoses such as nonruptured cerebral aneurysm, cerebral atherosclerosis, progressive vascular leukoencephalopathy, hypertensive encephalopathy or nonpyogenic sinus vein thrombosis which couldalsocontribute to the high prevalence of this diagnostic category.

**(vii)Other disorders of the nervous system**

The ICD-_10_ interval G_90_-G_99_ representing “Other disorders of the nervous system” included 5358 patients with two major subcategories displaying almost90% of the diagnoses: “**Disorders of autonomic nervous system” (G_90_ – 51.5%) and “Other disorders of brain” (G_93_ – 37.9%). The dominance of G_90_, an ICD-10 code rather rarely given by neurologists, may be attributed to the fact that diagnoses received from non-neurologists were also included in the study with the potential tendency to use this diagnostic category for more general complaints, e.g. dizziness, fainting, orthostatic hypotension, diarrhea, urinary incontinence or vaginal dryness. The high prevalence of subgroup G_93_ can be explained by theinvolvement ofconditions such as cerebral cysts, benign intracranial hypertension, unspecified encephalopathy, cerebral edema, and the categories of other specified and other unspecified disorders of the brain.**

**(viii)Demyelinating diseases of the central nervous system**

Multiple sclerosis is one of the most common causes of neurological disability in young people with the onset of the disease peaking between 20 and 40 years of age and with women being 2-3 times more frequently affected than men **[34]**.Epidemiological studiesconducted in Csongrád county in Hungary utilizing data from a local MS register resulted in an increasing crude MS prevalence in females from128.6 per 100 000 in 2013 to 149.3 per 100 000 in 2019 **[45,46]**.A recent study deriving data from healthcare administrative records showed MS being more prevalent in Hungary than previously thought with the sametendency in numbersincreasing from 150.8 per 100 000 to 179.5 per 100 000 between 2010 and 2015 among women **[20]**. FurtherstatisticsfromCentral Europe show a crude prevalence of MSup to 240 per 100000among females**[47,48]**.According to our study, over (80%) ofwomen (1751 patients)withinthe category of “Demyelinating diseases of the central nervous system”(G_35_-G_37_) received the diagnosis“Multiple sclerosis” (G_35_). The displayed relatively high prevalence of (0.2% )among all women with at least one laborduringthe study period could be attributed to the gender and age characteristics of the study population (i.e. women of childbearing age) and to the utilization of healthcare administrative data provided by all clinical specialties.

**Strengths and limitations**

Strengths of our study highlighted in the followings (1) the analysis of reports from a single-payer state health insurance system enabling the full coverage of national data; (2) long timeframe covering a time period from 2004 to 2016; (3) the above points resulted in a substantial number of cases; (4) such comprehensive data in a medical frontier are scarce and require further investigation. Also, our study hada few limitations:(1) the definition of “neurological” disorders beyond the G-category (“Diseases of the nervous system”) of ICD-_10_ has been determined by the authors; (2) neurological diagnoses were given by all clinical specialtieswithout being necessarily confirmed by neurologists; (3) medical reports submitted bygeneral practitioners were not involved in the study; (4) evaluation of the diagnoses was restricted by the use of 3-digit ICD-10 codes not allowing further, more sophisticated assessment; (5) having a defined time interval, only neurological diagnoses received within the given period were taken into account – also,the above principle was applied when assessing first pregnanciesduring the study period.

**Conclusions**

As the scientific literature is scarceon data from large populations concerning the wide range of neurological disorders, nationwide statistics enabling the optimization of prenatal care are desirable. As estimations on disease prevalence deriving data extracted from healthcare administrativereports have proven useful before, we applied this method in our study to gather information about neurological diagnoses received prior to first pregnanciesduring a specific 13-year interval. Main findings of ourstudy were the high prevalence of pregestational neurological disorders, the massive dominance of headachesfollowed by the rather nonspecific diagnosis of dizziness and giddiness, the relevance of nerve, nerve root and plexus disorders and epilepsy, and the importance of cerebrovascular disorders amongwomen of childbearing age. Further studies on specific disease categories including pregnancy outcomes, or neurological confirmation of the diagnoseswouldhelp us refine the characterization of this specific population.

**Acknowledgments**

We thank Prof. Dr. Daniel Bereczki (SemmelweisUniversity) for critically reviewing the manuscript and for providing valuable feedback. (include your institution in the acknowledgment text )

**References (cross check the reference and citation in the text)**

1. GranellaF, Sancez G, Pucci E, Nappi RE, Ghiotto N, Nappi G. Migraine with aura and reproductive life events: a case control study. Cephalalgia 2000;20:701-707.
2. Voskuhl R, Momtazee C. Pregnancy: effect on multiple sclerosis, treatment considerations, and breastfeeding. Neurotherapeutics2017;14:974-984.
3. Ducci RD, Lorenzoni PJ, Kay CSK, Werneck LC, Scola RH. Clinical follow-up of pregnancy in myasthenia gravis patients. NeuromusculDisord 2017;27:352-357.
4. The EURAP Study Group. Seizure control and treatment in pregnancy. Observations from the EURAP epilepsy pregnancy registry. Neurology 2006;66:354-360.
5. Camargo EC, Feske SK, Singhal AB. Stroke in pregnancy: an update. NeurolClin 2019;37:131-148.
6. Hamel J, Ciafaloni E. An update: myasthenia gravis and pregnancy. NeurolClin 2018;36:355-365.
7. Artama M, Braumann J, Raitanen J, Uotila J, Gissler M, Isojärvi J, et al. Women treated for epilepsy during pregnancy: outcomes from a nationwide population-based cohort study. ActaObstetGynecolScand 2017;96:812-820.
8. Jehan S, Auguste E, Zizi F, Pandi-Perumal SR, Gupta R, Attarian H, et al. Obstructivesleepapnea: women’sperspective. [J Sleep MedDisord](https://www.ncbi.nlm.nih.gov/pubmed/?term=Jehan+S%2C+et+al.+Obstructive+Sleep+Apnea%3A+Women%E2%80%99s+Perspective.)2016;3:1064.
9. Grossman TB, Govindappagari S, Dayal AK, Robbins MS. Delivery outcomes of patients with acute migraine in pregnancy: a retrospective study. Headache 2017;57:605-611.
10. vanAlebeek ME, de Vrijer M, Arntz RM, Maaijwee N, Synhaeve NE, Schoonderwaldt H, et al. Increased risk of pregnancy complications after stroke: the FUTURE study (Follow-Up of Transient Ischemic Attack and Stroke Patients and Unelucidated Risk Factor Evaluation). Stroke 2018;49:877-883.
11. Thomas SV, Jose M, Divakaran S, Sarma PS. Malformation risk of antiepileptic drug exposure during pregnancy in women with epilepsy: results from a pregnancy registry in South India. Epilepsia 2017;58:274-281.
12. O’Neal MA. Neurology of pregnancy: a case-oriented review. NeurolClin 2016;34:717-731.
13. Langer-Gould AM. Pregnancy and family planning in multiple sclerosis. Continuum (MinneapMinn) 2019;25:773-792.
14. Oberfrank F, Ajtay A, Bereczki D. Demand for neurological services in Central Eastern Europe: a 10-year national survey in Hungary. [Eur J Neurol](https://www.ncbi.nlm.nih.gov/pubmed/?term=Demand+for+neurological+services+in+Central+Eastern+Europe%3A+a+10-year+national+survey+in+Hungary) 2018;25:984-990.
15. International Statistical Classification of Diseases and Related Health Problems 10th Revision (Version: 2016). [Cited 2021 February 23]. In: World Health Organization Homepage [Internet]. Available from: https://icd.who.int/browse10/2016/en
16. Ajtay, A., Oberfrank, F., Bereczki, D. A kórháziadatlapokjelentéseinekalkalmazhatóságaepidemiológiaielemzésekreazischaemiáscerebrovascularisbetegségekpéldájaalapján [Applicability of hospital reports submitted for reimbursement purposes for epidemiological studies based on the example of ischemic cerebrovascular diseases]. Orv Hetil 2015;156:1540–1546.
17. Magyar M, Csepany E, Gyüre T, Ajtay A, Bereczki D, Oberfrank F, et al. Headache in Hungarian neurology outpatient services. Eur J Neurol 2016;23(Suppl 2):778.
18. Szatmári S Jr, Ajtay A, Bálint M, Takáts A, Oberfrank F, Bereczki D. Linking individual patient data to estimate incidence and prevalence of Parkinson's disease by comparing reports of neurological services and pharmacy prescription refills at a nationwide level. [Front Neurol](https://www.ncbi.nlm.nih.gov/pubmed/31275231?dopt=Abstract) 2019;10:640.
19. Szatmári S Jr, Ajtay A, Oberfrank F, Dobi B, Bereczki D. The prevalence of psychiatric symptoms before the diagnosis of Parkinson's disease in a nationwide cohort: a comparison to patients with cerebral infarction. PLoS One. 2020;15:e0236728.
20. Iljicsov A, Milanovich D, Ajtay A, Oberfrank F, Bálint M, Dobi B, et al. Incidence and prevalence of multiple sclerosis in Hungary based on record linkage of nationwide multiple healthcare administrative data. PLoS One. 2020;15:e0236432.
21. Vos T, Abajobir AA, Abate KH, Abbafati C, Abbas KM, Abd-Allah F, et al. Global, regional, and national incidence, prevalence and years lived with disability for 328 diseases and injuries for 195 countries, 1990-2016: a systematic analysis for the Global Burden of Disease Study 2016. Lancet 2017;390:1211-1259.
22. Stovner LJ, Nichols E, Steiner TJ, Abd-Allah F, Abdelalim A, Al-Raddadi RM, et al. Global, regional, and national burden of migraine and tension-type headache, 1990-2016: a systematic analysis for the Global Burden of Disease Study 2016. Lancet Neurol 2018;17:954-976.
23. Stovner LJ, Hagen K, Jensen R, Katsarava Z, Lipton RB, Scher AI, et al. The global burden of headache: a documentation of headache prevalence and disability worldwide. Cephalalgia 2007;27:193-210.
24. Kroenke K, Mangelsdorff AD. Common symptoms in ambulatory case: incidence, evaluation, therapy, and outcome.Am J Med 1989;86:262-266.
25. Neuhauser HK. The epidemiology of dizziness and vertigo. HandbClinNeurol 2016;137:67-82.
26. Newman-Toker DE, Hsieh YH, Camargo CA Jr, Pelletier AJ, Butchy GT, Edlow JA. Spectrum of dizziness visits to US emergency departments: cross-sectional analysis from a nationally representative sample.Mayo Clin Proc 2008;83:765-775.
27. Doughty CT, Bowley MP. Entrapment neuropathies of the upper extremity. Med Clin North Am 2019;103:357-370.
28. Stevens JC, Sun S, Beard CM, O’Fallon WM, Kurland LT. Carpal tunnel syndrome in Rochester, Minnesota, 1961 to 1980. Neurology 1988;38:134-138.
29. Kincaid JC. Upper extremity neuropathies. HandbClinNeurol 2019;161:197-205.
30. Burton CL, Chen Y, Chesterton LS, van der Windt DA. Trends in the prevalence, incidence and surgical management of carpal tunnel syndrome between 1993 and 2013: an observational analysis of UK primary care records. BMJ Open 2018;8:e020166.
31. Macfarlene TV, Blinkhorn AS, Davies RM, Kincey J, Worthington HV. Oro-facial pain in the community: prevalence and associated impact. Community Dent Oral Epidemiol 2002;30:52-60.
32. Weiss AL, Ehrhardt KP, Tolba R. Atypical facial pain: a comprehensive evidence-based review. Curr Pain Headache Rep 2017;21:8.
33. De Toledo IP, Conti Réus J, Fernandes M, Porporatti AL, Peres MA, Takaschima A, et al. Prevalence of trigeminal neuralgia: a systematic review. J Am Dent Assoc 2016;147:570-576.e2.
34. Oh J, Vidal-Jordana A, Montalban X. Multiple sclerosis: clinical aspects. CurrOpinNeurol 2018;31:752-759.
35. Godazandeh K, Sosa SM, Wu J, Zakrzewska JM. Trigeminal neuralgia: comparison of characteristics and impact in patients with or without multiple sclerosis. MultSclerRelatDisord 2019;34:41-46.
36. Hurwitz EL, Randhawa K, Yu H, Côté P, Haldeman S. The Global Spine Care Initiative: a summary of the global burden of low back and neck pain studies. Eur Spine J 2018;27(Suppl 6):796-801.
37. Peitersen E. Bell’s palsy: the spontaneous course of 2,500 peripheral facial nerve palsies of different etiologies. ActaOtolaryngolSuppl 2002;549:4-30.
38. Farmen AH, Grundt JH, Tomson T, Nakken KO, Nakling J, Mowinchel P, et al. Age-specific birth rates in women with epilepsy: a population-based study. BarinBehav 2016;6:e00492.
39. Pennell PB, French JA, Harden CL, Davis A, Bagiella E, Andreopoulos E, et al. Fertility and birth outcomes in women with epilepsy seeking pregnancy. JAMA Neurol 2018;75:962-969.
40. Gooneratne IK, Wimalaratna S. Update on management of epilepsy in women for the non-neurologist. Postgrad Med J 2016;92:554-559.
41. Truelsen T, Piechowski-[Jóźwiak](https://pubmed.ncbi.nlm.nih.gov/?term=Piechowski-J%C3%B3%C5%BAwiak+B&cauthor_id=16796582) B, Bonita R, Mathers C, BogousslavskyJ, Boysen G. Stroke incidence and prevalence in Europe: a review of available data. Eur J Neurol 2006;13:581-598.
42. Dhingra R, Vasan RS. Age as a cardiovascular risk factor. Med Clin North Am 2012;96:87-91.
43. Martin JA, Hamilton BE, Osterman MJK. Births in the United States, 2016. NCHS Data Brief 2017;287:1-8.
44. Statistical Bulletin: Live Births in England and Wales by Characteristics of Mother 1: 2013. [Cited 2021 February 23]. In: Office for National Statistics Homepage [Internet]. Available from: https://www.ons.gov.uk/peoplepopulationandcommunity/birthsdeathsandmarriages/livebirths/bulletins/livebirthsinenglandandwalesbycharacteristicsofmother1/2014-10-16
45. Zsiros V, Fricska-Nagy Z, Füvesi J, Kincses ZT, Langane É, Paulik E, et al. Prevalence of multiple sclerosis in Csongrád County, Hungary. ActaNeurolScand 2014;130:277–282.
46. Biernacki T, Sandi D, Fricska-Nagy Zs, Kincses ZT, Füvesi J, Laczkó R, et al. Epidemiology of multiple sclerosis in Central Europe, update from Hungary. Brain Behav 2020;10:e01598.
47. Höer A, Schiffhorst G, Zimmermann A, Fischaleck J, Gehrmann L, Ahrens H, et al. Multiple sclerosis inGermany: dataanalysis of administrativeprevalence and healthcaredeliveryinthestatutoryhealthsystem. BMC Health Serv Res 2014;14:381.
48. Bezzini D, Battaglia MA. Multiple sclerosis epidemiology in Europe. AdvExp Med Biol 2017;958:141-159.

**Supporting information**

**S1 File.**
