# Supplementary material for: Pregestational neurological disorders among women of childbearing age—Nationwide data from a 13-year period in Hungary
Source: PLoS One. 2022 Sep 21;17(9):e0274873. doi: 10.1371/journal.pone.0274873 (PMC9491540; doi:10.1371/journal.pone.0274873)
Supplement: Attachment — Submitted filename: Plos editor.docx [file pone.0274873.s005.docx]

Dear Chief Editor

Thank you for very much for choosing academic editor for the present research paper entitled “Pregestational neurological Disorders among Women of Child bearing age – Nationwide data from a 13-year period in Hungary” I have carefully re-reviewed and highlighted the certain points in the reviewer track . Requested the editor to communicate the author for necessary changes in the submitted research paper for final decision .

Best Regards

Dr Basavarajaiah DM

Academic editor
