# Supplementary material for: Pregestational neurological disorders among women of childbearing age—Nationwide data from a 13-year period in Hungary
Source: PLoS One. 2022 Sep 21;17(9):e0274873. doi: 10.1371/journal.pone.0274873 (PMC9491540; doi:10.1371/journal.pone.0274873)
Supplement: Attachment — Submitted filename: Response to Reviewers.docx [file pone.0274873.s008.docx]

**János Szentágothai Doctoral School of Neurosciences**

Semmelweis University

Chairman: Prof. Dániel Bereczki MD. PhD. DHAS

H-1085 Budapest, Üllői út 26.

Tel.: +36-1-459-1500

Tel.: +36-1-459-1500

Budapest, September 3, 2022

**Dr. D. M. Basavarajaiah**

**Academic Editor**

**Public Library of Science One**

Dear Editor,

thank you for the additional comments provided on our manuscript Bereczki D Jr. et al: **Pregestational neurological disorders among women of childbearing age – nationwide data from a 13-year period in Hungary** submitted with the intention to publish it in the journal **PLOS ONE**.

Please, find our responses to the comments:

***(i) “Throughout - clarify that GP is not considered a specialty; diagnoses were taken from secondary care practitioners. (to my understanding.)”***

For the sake of clarity, we reworded the concerned parts of the manuscript:

In the *Materials and methods* section, the updated wording is as follows: *“To exclude non-clinical specialty areas (e.g. laboratory diagnostics, diagnostic imaging, physiotherapy, psychology, etc.), only diagnoses which had been confirmed by secondary care clinical specialties were involved in the study by the use of specific clinical specialty codes applied in Hungary. It is to be noted that primary care reports submitted by general practitioners were not included in the database.”*

In the *Strengths and limitations* section, the updated wording is as follows: *“(3) the dataset contained diagnoses given by secondary care specialists, however medical reports submitted by general practitioners were not involved in the study”*

***(ii) All of the grammatical suggestions have been implemented in the updated manuscript.***

We hope that after considering our revisions and comments, you will find our manuscript worth to consider it for publication.

Sincerely yours,

Dániel Bereczki Jr., MD

Corresponding author

E-mail: bereczki.daniel@hotmail.com
