# Supplementary material for: Pregestational neurological disorders among women of childbearing age—Nationwide data from a 13-year period in Hungary
Source: PLoS One. 2022 Sep 21;17(9):e0274873. doi: 10.1371/journal.pone.0274873 (PMC9491540; doi:10.1371/journal.pone.0274873)
Supplement: Attachment — Submitted filename: reviewer comment 2.docx [file pone.0274873.s002.docx]

Dear author,

I would like to acknowledge that, I have reviewed your paper titled “**Pregestational neurological disorders among women of childbearing age - nationwide data from a 13-year period in Hungary submitted by Dániel Bereczki”** It is a very important research topic and findings will be deeply help us to assess the clinical indicators at micro level , serving as guide for neurologists and clinicians and also help us to implementation of health policy at national level. The rationality and selection of topic is very excellent (PONE-D-21-09199)

Below are a few comments that the authors can consider.

(i)The author unable to describe the research gap and rationality is not up to the mark, the practical utility of the research is well planned and derived properly. In Methodological section, I have not seen , what are the tests used for assessing the patients and selection of concomitant variables.

(ii) In result part, the research hypothesis is not tested by relevant statistical methods .The flow of resulted part is not fulfilled our journal criteria. Plz repharse the sentence and describe the result part by using accurate estimation of variables to be tested by statistical methods.

(iii) Discussion and conclusion part is not fulfilled our journal criteria-Major revision should be necessary by the author

Decision of the research paper: Major revision and resubmission, because topic is more useful for the scientific community

Requested the Author, plz Rephrase the above limitation and comments.
