# Supplementary material for: Pregestational neurological disorders among women of childbearing age—Nationwide data from a 13-year period in Hungary
Source: PLoS One. 2022 Sep 21;17(9):e0274873. doi: 10.1371/journal.pone.0274873 (PMC9491540; doi:10.1371/journal.pone.0274873)
Supplement: Attachment — Submitted filename: reviewer comment 2 (1).docx [file pone.0274873.s006.docx]

Dear editor

**Title of the research Paper : Pre gestational neurological disorders among women of childbearing age - nationwide data from a 13-year period in Hungary submitted by Dániel Bereczki” PONE-D-21-09199**

I have carefully reviewed it, all corrections were done by the author are very excellent. The revised paper is more relevant and more applicable to present neurological manifestation scenarios for implementing clinical guidance about the affected population.  The revised paper is accepted for publication inclusion with adding recent literature citation in the discussion part

**Dr Basavarajaiah DM**

**Ph.D**

**Academic  editor**
